# Feasibility of ventilator-assisted tubeless anesthesia for video-assisted thoracoscopic surgery

**DOI:** 10.1097/MD.0000000000034220

**Published:** 2023-07-14

**Authors:** Hyo-Jin Kim, Myeongjun Kim, Byungjoon Park, Yong-Hee Park, Se-Hee Min

**Affiliations:** a Department of Anesthesiology and Pain Medicine, Chung-Ang University Gwangmyeong Hospital, Chung-Ang University College of Medicine, Gyeonggi-do, Republic of Korea; b Department of Anesthesiology and Pain Medicine, Chung-Ang University Hospital, Chung-Ang University College of Medicine, Dongjak-gu, Seoul, Republic of Korea; c Department of Thoracic and Cardiovascular Surgery, Chung-Ang University Hospital, Chung-Ang University College of Medicine, Dongjak-gu, Seoul, Republic of Korea.

## Abstract

General anesthesia providing one-lung ventilation (OLV) with double-lumen endotracheal intubation has been considered inevitable for thoracic surgery. However, with the recent trend of less invasive surgical technique and enhanced recovery after surgery, tubeless anesthesia has been performed in various thoracic surgeries. The aim of this study was to establish a feasible and safe strategy of ventilator-assisted tubeless anesthesia in video-assisted thoracoscopic surgeries (VATS) based on single-institution experiences. We retrospectively reviewed the medical records of patients who underwent tubeless VATS from November 2019 to December 2021. Perioperative anesthetic and surgical variables as well as complications were reported. Seventeen patients with a median age of 29 and American Society of Anesthesiologists physical status I to II underwent video-assisted pulmonary wedge resection under monitored anesthesia care (MAC) using propofol and remifentanil. Mechanical ventilation was applied in synchronized intermittent mandatory ventilation with pressure support mode through facemask if respiratory support was required. During the operation, none of the patients showed hypoxemia or involuntary movement interfering operation. No patients were converted to general anesthesia or open thoracotomy unintentionally. All patients were discharged on median 2 days postoperatively without complications. Ventilator-assisted tubeless VATS is a feasible and safe option in low-risk patients undergoing video-assisted pulmonary wedge resection.

## 1. Introduction

General anesthesia with one-lung ventilation (OLV) using a double-lumen endotracheal tube or an endobronchial blocker has traditionally been recognized mandatory for thoracoscopic operations. Although general anesthesia with OLV has an indisputable advantage of providing optimal conditions for surgical manipulation, it is associated with complications including airway trauma, ventilator-induced lung injury and residual neuromuscular blockade.^[1–5]^

With technical advances, minimally invasive surgery and anesthesia are evolving in the field of thoracic surgery.^[6]^ Tubeless anesthesia in video-assisted thoracoscopic surgeries (VATS) with intercostal nerve block (ICNB) or thoracic plane blocks such as thoracic paravertebral block (TPVB) or thoracic epidural block, has been revealed to have clinical advantages over general anesthesia.^[7–9]^ In addition, the emergence of the concept of enhanced recovery after surgery facilitated a tubeless approach in thoracic operations, which has been recently reported.^[10–13]^

The aim of this study was to establish a feasible and safe strategy of tubeless VATS based on single-institution experiences.

## 2. Materials and Methods

### 2.1. Ethical approval

This study was approved by the Institutional Review Board of Chung-Ang University Hospital (IRB No. 2208-009-19431; Principal investigator, Se-Hee Min; Date of registration, August 22nd, 2022). The requirement for informed consent of individual patients was waived since we used deidentified administrative claims data.

### 2.2. Study population

Since 2019, our hospital has been performing tubeless VATS on selected patients aged ≥ 20 years who consent to monitored anesthesia care (MAC) regarding surgical and anesthetic safety. Surgical indications of tubeless VATS included VATS bullectomy, VATS pleural or lung biopsy, and VATS wedge resection. In these patients, the anesthesiologists evaluated risk factors associated with emergent conversion to general anesthesia, including predicted difficult airway, risk of pulmonary aspiration or coagulopathy, history of sleep apnea, previous thoracic surgery with potential pleural adhesion, and hemodynamic instability.

Based on this protocol, the electronic medical records of 17 patients who underwent tubeless VATS from November 2019 to December 2021 were reviewed retrospectively.

### 2.3. Anesthetic management

Before inducing MAC, we confirmed complete preparations at the patient bedside in case of unintentional conversion to general anesthesia: video laryngoscope, 35-Fr and 37-Fr left-sided double-lumen endotracheal tubes, endotracheal tubes with an inner diameter of 7.0 to 8.0 mm, supraglottic airway, stylet, an endobronchial blocker, a fiberoptic bronchoscope (LF-GP; Olympus Optical Co., Tokyo, Japan), a suction catheter, lubrication jelly and rocuronium.

After arriving at the operating room, patients were monitored using pulse oximetry, noninvasive blood pressure, electrocardiography, heart rate and bispectral index (A-2000 XP; Aspect Medical Systems, Newton, MA), which were recorded every 5 minutes until the end of surgery.

**Figure 1.**
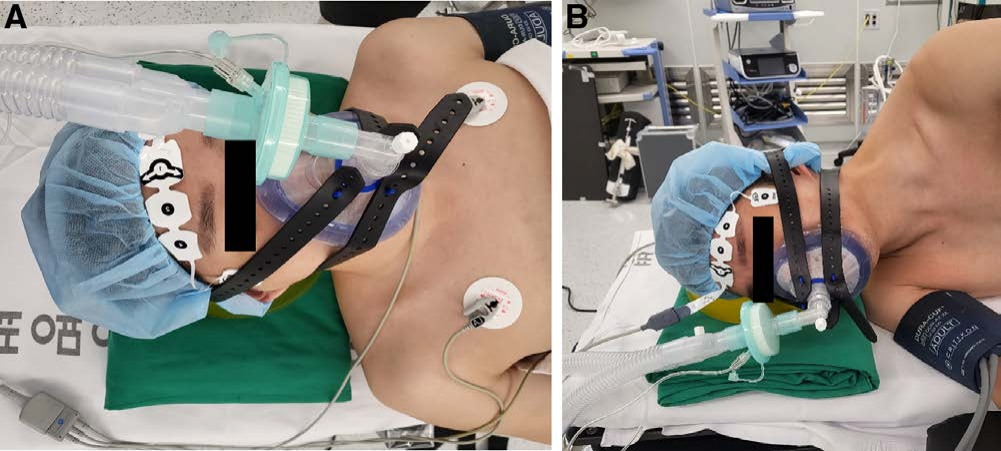
Tubeless anesthesia applied to a patient in supine (A) and lateral decubitus position (B).

Without premedication, 100% oxygen was supplied at 10 L/min through a transparent facemask for preoxygenation. Following preoxygenation, MAC was induced with total intravenous anesthesia using target-controlled infusion (Base Primea; Fresenius Vial Brézins, France) of propofol (effect-site concentration, 0.8–2 µg/mL) and remifentanil (effect-site concentration, 0.8–2 ng/mL). After confirming loss of consciousness and bispectral index of <60, a lubricated nasopharyngeal airway was inserted gently into the nostril to secure airway without causing discomfort to the patients. Then, oxygen flow rate was adjusted to 4 L/min with 100% oxygen. The facemask was closely attached to the patient face and fixed with rubber band in case of requiring mechanical ventilatory support in lateral decubitus position (Fig. 1A). The patient spontaneous ventilation was monitored with capnogram using a side stream capnometer of mechanical ventilator (Dräger, Lübeck, Germany).

After lateral decubitus positioning, the patient head was kept in a neutral position on the headrest. Both elbows were slightly flexed on the armrests, and a roll was placed just caudal to the dependent axilla (Fig. 1B). Then, the patient was slightly flexed at the mid-chest level to expose a sufficient surgical field.

During the surgery, the concentrations of propofol and remifentanil were adjusted and maintained in range of bispectral index of 40 to 60. Mechanical ventilation was applied in synchronized intermittent mandatory ventilation with pressure support mode if respiratory support was required. If the patient showed involuntary movements, midazolam 1 to 2 mg was administered intravenously. Hypotension was defined as more than 30% decrement from baseline systolic blood pressure or when the systolic blood pressure <90 mm Hg, which was treated with 5 mg of ephedrine or 50 to 100 mcg of phenylephrine administration. Bradycardia was defined as more than 30% decrease in heart rate compared to baseline or when the heart rate showed <45 beats/min, which was treated by 0.25 to 0.5 mg of atropine or 0.2 mg of glycopyrrolate administration.

Conversion to general anesthesia was planned in the following cases: moderate to severe bleeding, unexpected extensive pleural adhesions, hypoxemia defined as oxygen saturation <90%, hemodynamic instability, such as uncontrolled hypotension, bradycardia, or intractable arrhythmia, involuntary or deep respiratory movements delaying surgery. The anesthesiologist in charge planned immediate single lumen endotracheal intubation using a video-laryngoscope in lateral decubitus position if conversion to general anesthesia was needed. An endobronchial blocker and a fiberoptic bronchoscope were prepared so that the operation could be performed under OLV.

Propofol and remifentanil were gradually discontinued during the skin suture to awake the patient at the end of the surgery.

### 2.4. Surgical technique

All patients underwent 3-port VATS using the same technique by a single thoracic surgeon. An incision was created in the fourth or fifth intercostal space after infiltrating local anesthetics (2% lidocaine plus 0.5% bupivacaine) from the skin to the parietal pleura. After opening the parietal pleura, the lung was collapsed by an iatrogenic pneumothorax which have caused tachypnea temporarily. ICNB was performed along the lower borders of the third to eighth ribs using a 1-mL mixture containing 2% lidocaine and 0.5% bupivacaine (1:1) for postoperative pain control. After pulmonary resection, a 24-Fr chest tube was placed through the lower incision line at the completion of surgery.

### 2.5. Postoperative management

After the operation, the patient was transferred to the postanesthetic care unit (PACU). The patient was asked to deeply breathe and encouraged to cough for re-expanding collapsed lungs after fully awake. Supplemental oxygen was applied via the facemask during PACU stay to maintain adequate oxygen saturation. The patient was ventilated in room air when oxygenation was not required.

During the PACU stay, postoperative pain was immediately assessed, and 50 µg of fentanyl was administered if required. Postoperative nausea or vomiting was also recorded, and ramosetron hydrochloride was prepared for treatment. Patients with a modified Aldrete score of 9 to 10 were transferred to the general ward from the PACU.

Postoperative pain score was evaluated and treated with ketorolac tromethamine or acetaminophen administration for 2 days postoperatively. A chest X-ray radiograph was obtained for 2 days after the surgery to detect postoperative pulmonary complications. The chest tube was removed when drainage amounts were <200 to 250 mL without air leakage.

### 2.6. Data collection and statistical analysis

Data including patient demographics, intraoperative variables, including vital signs and surgical results, and postoperative variables were collected from the institutional database, anesthesia and surgical notes, and nursing records.

We recorded amounts of anesthetic agents, additional sedatives, inotropes, estimated blood loss and fluids. Intraoperative vital signs, including blood pressure, heart rate, oxygen saturation and bispectral index were measured every 5 minutes. The lowest oxygen saturation during surgery were also recorded.

Postoperative pain score was assessed with a visual analogue scale (VAS) immediately after surgery until the second postoperative day. Rescue analgesics used for 2 days postoperatively were recorded. We also assessed postoperative complications, including nausea, vomiting, shivering as well as cardiorespiratory status requiring treatment with inotropes or supplemental oxygen. A chest X-ray radiograph was obtained immediately, and 24 and 48 hours after surgery, which were evaluated by chest radiologists. We checked time for anesthesia and surgery, and the length of stay in PACU and hospital.

Statistical analyses were performed using IBM SPSS Statistics for Windows software (version 21.0; IBM Corp., Armonk, NY). For continuous variables, the Shapiro–Wilk test was performed for normality test. Depending on the normal distribution, continuous variables are analyzed with the Mann–Whitney *U* test and independent *t* test, respectively. Continuous variables are expressed as mean (SD) or median (interquartile range). Categorical variables were analyzed with the chi-square test and described as frequency and percentage.

## 3. Results

The characteristics of the patients, anesthesia, and surgery are shown in Table 1. Seventeen patients underwent tubeless anesthesia for VATS. There were 13 males and 4 females, and the median age was 29 (18, 44) years, whereas the oldest was 90 years old. The mean body mass index was 20.5 (2.0) kg/m^2^.

**Table 1:** Characteristics of patient, anesthesia, and surgery.

Variables	
Age (yr)	29 (18–44)
Male/Female	13/4
Height (cm)	172.7 ± 9.9
Weight (kg)	61.5 ± 8.6
Body mass index (kg/m^2^)	20.5 ± 2.0
ASA physical status (I/II)	2/15
Medical conditions (hypertension/diabetes/COPD/stroke)	2/1/0/0
Pulmonary function test	
DL_CO_ (mL/mm Hg/min)	16.4 ± 1.8
FEV_1_ (L)	2.51 ± 2.8
FEV_1_/FVC (%)	76.4 ± 2.1
Diagnosis	
Pneumothorax	12
Solitary pulmonary nodule	4
Interstitial pneumonia	1
Side of surgery (left/right)	9/8
Type of surgery	
Bullectomy	12
Wedge resection	5
Amount of anesthetic drugs	
Propofol (mg)	400 (300–400)
Remifentanil (mcg)	400 (400–600)
Inotropic requirement	0
Duration of surgery (min)	35 (30–45)
Duration of anesthesia (min)	65 (60–70)

Values are presented as numbers, median (interquartile range), or mean ± standard deviation.

ASA = American Society of Anesthesiologists, DL_CO_ = diffusing capacity of the lung for carbon monoxide, FEV_1_ = forced expiratory volume in 1 second, FVC = forced vital capacity.

Twelve patients who were diagnosed with pneumothorax underwent thoracoscopic bullectomy. Four patients who were diagnosed with solitary pulmonary nodules underwent thoracoscopic wedge resection, whereas 1 patient who was diagnosed with interstitial pneumonia underwent lung biopsy. The mean operation time was 34.7 (9.8) min and anesthesia time was 62.9 (11.0) min.

**Figure 2.**
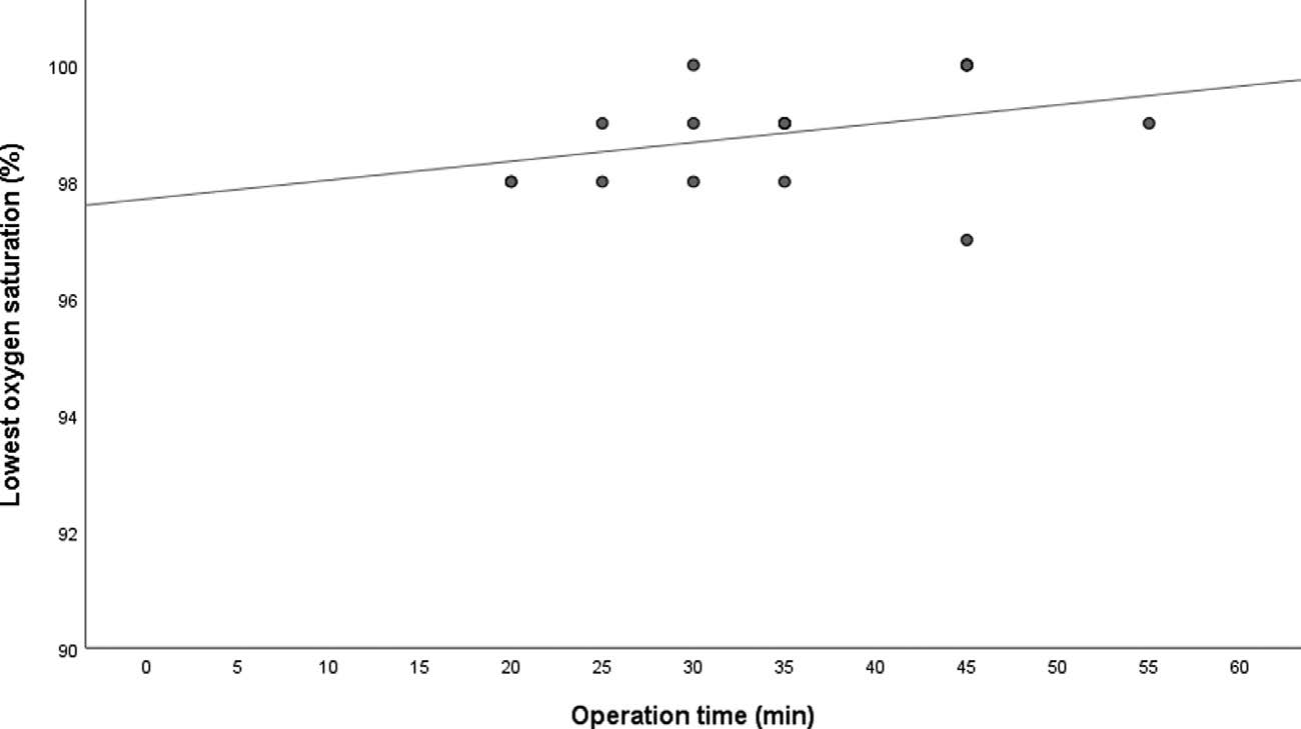
The relationship between operation time and lowest oxygen saturation during operation (n = 17).

During lateral positioning and preparing for surgery, 10 patients showed involuntary movements, which were controlled with 1 to 2 mg of midazolam administration. Since deep sedation was maintained in range of bispectral index of 40 to 60, mechanical ventilatory support was applied to all patients during the surgery. As a result, none of the patients showed inadequate movements requiring additional sedatives to proceed operation (Table 2). Intraoperative oxygen saturation was maintained between 98% and 100% in all patients except for 1 patient who showed the value of at least 97% (Fig. 2). Mean blood pressure was maintained between 61.9 and 96.1 mm Hg. The average heart rate was maintained in the range of 60.5 to 80.5 beats/min. None of the patients showed unexpected extensive pleural adhesions or any other limitations obstructing the surgical field. Intraoperative blood loss did not exceed 20 mL in either patient. All patients safely underwent tubeless VATS without conversion to general anesthesia or open thoracotomy (Table 2).

**Table 2:** Intraoperative complications associated with tubeless VATS.

Variables	
hypoxemia	0
Involuntary movement	10
Amount of additive midazolam (mg)	1.8 (1.0–2.0)
Amount of additive analgesics	0
Coughing	0
Interference of surgical field	0
Conversion to general anesthesia	0
Conversion to open thoracotomy	0

Values are presented as numbers, or median (interquartile range).

VATS = video-assisted thoracoscopic surgeries.

Postoperatively, 7 patients required analgesics for pain control at the PACU. The median VAS score during the PACU stay was 3, which decreased to 2 on the first postoperative day. Only 1 patient required supplemental oxygen of 2 L/min via a nasal prong when transferred to the general ward from the PACU, and changed stepwise to room air in an hour. In the general ward, all patients were able to resume water and food intake within 2 hours. Among 17 patients, 3 showed subsegmental atelectasis in the operative lung immediately and 1 day after the surgery, which resolved on day 2 postoperatively. None of the patients showed other postoperative pulmonary complications including lung infiltration (Table 3). All patients were discharged from the hospital within 3 days after surgery, except one who required additional medical treatment for interstitial pneumonia previously diagnosed.

**Table 3:** Postoperative outcomes associated with anesthesia and surgery.

Variables	PACU	POD 1	POD 2
Applying Supplemental oxygen	1	0	0
VAS	3 (3–6)	2 (2–4)	NR
Amount of additional analgesics			
Fentanyl (mcg)	50	0	0
Ketorolac (mg)	0	30	0
Acetaminophen (mg)	0	500	0
Nausea/vomiting	0	0	0
Chest X-ray			
Atelectasis	3	3	0
Lung infiltration	0	0	0
PACU stay (min)	35 (30–45)		
Chest tube removal (POD)	1 (1–3)		
Discharge after surgery (POD)	2 (2–3)		
Hospital stay (d)	7 (6–8)		

Values are presented as numbers, or median (interquartile range).

NR = not recorded, PACU = post-anesthesia care unit, POD = postoperative day, POD 1 = The first postoperative day, POD 2 = the second postoperative day, VAS = visual analogue scale.

## 4. Discussion

Intubating anesthesia providing OLV has been considered mandatory for VATS over decades. However, to avoid adverse effects of general anesthesia and endotracheal intubation, VATS under MAC or awake surgery with locoregional anesthesia has recently been tried in selected patients.^[2–5,14–21]^ Tubeless anesthesia appears to be a new trend that could replace intubated anesthesia; however, it also has inherent risks, such as unexpected patient movement, hypoxia, or hypercapnia. In this aspect, our strategy for ventilator-assisted tubeless anesthesia can be a feasible and safe alternative to conventional general anesthesia for thoracoscopic pulmonary wedge resection.

Tubeless VATS was initially reported in 2004. Pompeo et al^[22]^ demonstrated surgical feasibility as well as patients’ great satisfaction score and short hospital stay of sole thoracic epidural anesthesia in VATS. Thereafter, tubeless VATS has been performed from minor operations, such as thoracoscopic lung biopsy, to major operations, such as lobectomy and carinal resection, which were reported in a few case reports and large-volume experiences.^[7,14,16,18–21,23–25]^ Although still uncommon, there are meta-analyses showing better perioperative outcomes of tubeless VATS with reduced operating room time, postoperative complications, such as nausea and vomiting, shorter postoperative fasting, and hospital stay while providing equivalent visual conditions for surgery compared with VATS under general anesthesia.^[8,26,27]^

The main advantage of emerging tubeless VATS is to minimize complications following general anesthesia and OLV by maintaining spontaneous breathing throughout the operation. Although the conventional method using DLT guarantees lung separation and surgical vision, it is associated with complications such as airway trauma, ventilator-induced lung injury and residual neuromuscular blockade.^[2–5]^ Due to the size and rigidity of a DLT, airway injuries vary from mild transient symptoms, such as sore throat, to fatal sequelae, such as vocal cord paralysis, tracheal stenosis and airway rupture.^[28]^ Tubeless VATS eliminates the risk of intubation-related complications as well as the risk of postoperative pulmonary complications associated with mechanical OLV. In addition, by avoiding the use of neuromuscular blocking agents, the risk of respiratory muscle weakness and diaphragmatic dysfunction can be eliminated, causing atelectasis and impairment of protective airway reflexes postoperatively.^[4]^ Furthermore, by not inducing intrapulmonary shunts, the risk of cerebral desaturation and post-thoracotomy delirium can be reduced.^[29,30]^

Considering such intubating general anesthesia-related events, tubeless VATS is expected to be beneficial in patients, especially the elderly with high risk of postoperative pulmonary complications or cognitive dysfunction.^[31]^ Katlic et al^[32]^ reported that the safety and efficacy of tubeless VATS under locoregional anesthesia with sedation in the geriatric patients aged 80 years and older. Another retrospective study also showed safely performed lung biopsies with tubeless anesthesia in patients with American Society of Anesthesiologists physical status of III and IV.^7^ In our study, we applied tubeless VATS in patients aged 80 and 90 years. In 2 patients, surgery was completed without any complications, and they were discharged from the hospital on the second and third days postoperatively.

Postoperative pain control by locoregional anesthesia is one of the crucial factors facilitating tubeless VATS.^[33]^ TPVB, one of the most commonly used techniques, provides analgesia to the chest wall and corresponding dermatomes, thereby supporting surgical procedures under MAC as well as postoperative pain relief.^[11,12]^ Erector spinae plane block is also one of the locoregional anesthetic techniques, which provides anesthesia to the posterior thoracic wall by injecting local anesthetics between the erector spinae muscle and the underlying ribs.^[34]^ This technique has become popular, since it covers wider areas compared to TPVB. In our cases, we applied ICNB. Since surgeons inject local anesthetics for ICNB during surgery, ICNB may cause fewer side effects compared to other nerve blocks, along with effective postoperative pain control.^13^

Despite these advantages, there are challenging issues on tubeless VATS. First, there is a risk of hypoventilation since patients can breathe spontaneously under deep sedation for thoracoscopic surgery.^[35]^ To maintain adequate ventilation without involuntary movement interfering surgery, we applied respiratory support in synchronized intermittent mandatory ventilation with pressure support mode with deep sedation of bispectral index <60. Although bispectral index <60 is the target level for general anesthesia, we maintained this level of sedation to satisfactorily control possible disturbing situations in MAC compared to general anesthesia. There have been several studies applying supportive oxygenation via laryngeal mask airways or high-flow nasal cannulas in tubeless VATS.^[36–38]^ However, these methods have difficulties in assisting ventilation when respiratory depression develops in the lateral decubitus position. Therefore, we applied a fitted facial mask to the patient with ventilatory support to secure patient oxygenation under deep sedation. As a result, none of the patients showed hypoxemia under oxygen saturation of 97% during surgery and required immobilization to proceed operation. Secondly, there are surgery-related risks of emergent conversion to general anesthesia, such as massive bleeding or severe pleural adhesions.^[9,39]^ Patient-related risk factors, such as the patient discomfort and pain are also important concerns throughout tubeless VATS. Major thoracoscopic surgeries usually require lung traction and hilar manipulation, which can trigger the cough reflex and impeded movements during the surgery. Therefore, additional regional anesthesia, such as thoracic epidural anesthesia and vagal block, should be performed for safe surgical procedures. For this reason, most of the tubeless VATS cases were reported in limited experiences undergoing minor thoracoscopic surgeries.^[40–42]^ We also performed tubeless VATS on patients who underwent pulmonary wedge resection with minimal surgical risk of unexpected conversion to general anesthesia. However, with ongoing trends toward less invasive surgical techniques and enhanced recovery after surgery, it appears to be inevitable that anesthetic management should follow major thoracic surgeries. For this reason, tubeless VATS requires experienced thoracic surgeons and anesthesiologists with thorough preparation and vigilance for immediate endotracheal intubation in lateral decubitus position. Above all, patients should be selected carefully, and sufficient explanation should be given to the patients.

Our study has several limitations. First, this is a single-center experience of 17 patients without the control group. Due to a small number of patients included, there are insufficient evidence for primary outcomes such as the incidence of conversion to general anesthesia and the mortality rate. Secondly, this is a retrospective medical chart review. Due to a small sample size, statistical power is not robust. Thirdly, this study enrolled patients with American Society of Anesthesiologists physical status I or II with preserved functional lung capacities. Therefore, further prospective studies with a large sample size are warranted in minor to major thoracoscopic surgeries.

## 5. Conclusions

In conclusion, tubeless anesthesia with intermittent ventilator support can be a feasible and safe option for video-assisted pulmonary wedge resection. Through preparation for emergent conversion to general anesthesia, our anesthetic strategy is expected to be beneficial in patient postoperative complications for thoracoscopic surgeries.

## Acknowledgments

This research was supported by the Chung-Ang University Research Scholarship Grants in 2021.

## Author contributions

**Conceptualization:** Yong-Hee Park, Se-Hee Min.

**Data curation:** Myeongjun Kim, Byungjoon Park, Yong-Hee Park, Se-Hee Min.

**Formal analysis:** Hyo-Jin Kim.

**Investigation:** Se-Hee Min.

**Methodology:** Se-Hee Min.

**Supervision:** Se-Hee Min.

**Visualization:** Se-Hee Min.

**Writing – original draft:** Hyo-Jin Kim, Se-Hee Min.

**Writing – review & editing:** Hyo-Jin Kim, Yong-Hee Park, Se-Hee Min.
